# Mind-wandering content differentially translates from lab to daily life and relates to subjective stress experience

**DOI:** 10.1007/s00426-019-01275-2

**Published:** 2019-12-12

**Authors:** Roman Linz, Reena Pauly, Jonathan Smallwood, Veronika Engert

**Affiliations:** 1 Research Group “Social Stress and Family Health”, Max Planck Institute for Human Cognitive and Brain Sciences, Stephanstr. 1a, 04103 Leipzig, Germany; 2 Department of Psychology, University of York, York, UK; 3 Department of Social Neuroscience, Institute of Psychosocial Medicine and Psychotherapy, University Hospital, Friedrich-Schiller University, Jena, Germany

## Abstract

Experience and thoughts that are unrelated to the external surroundings are pervasive features of human cognition. Research under the rubric of mind-wandering suggests that such internal experience is context-dependent, and that the content of ongoing thought differentially influences a range of associated outcomes. However, evidence on how the extent of mind-wandering and its content translate from the laboratory to daily life settings is scarce. Furthermore, the relationship between such patterns of thought with markers of stress in daily life remains underexplored. In the current study, we examined multiple aspects of mind-wandering of ninety-three healthy participants (47 women, 25.4 ± 3.9 years) in both the laboratory and daily life and explored two questions: (a) how are mind-wandering extent and content correlated across both settings, and (b) what are their relationships with subjective stress and salivary cortisol levels in daily life? Our results suggest that the extent of off-task thinking is not correlated across contexts, while features of content—i.e., social, future-directed and negative thought content—robustly translate. We also found that daily life subjective stress was linked to more on-task, negative, and future-directed thinking, suggesting stress was linked with the need to act on personally relevant goals. Based on these results we speculate that differences in the links between stress and ongoing thought in daily life may be one reason why patterns of thinking vary from lab to everyday life. More generally, these findings underline the need to consider both context and content in investigating mind-wandering and associated features of subjective experience, and call for caution in generalizing laboratory findings to participants’ daily lives.

## Introduction

Disengaging from external stimulation and letting the mind wander from the here-and-now is a common phenomenon in everyday life. In fact, humans tend to engage in thoughts that are at least partially unrelated to their current task or environment for up to half of their conscious time (Kane et al., 2007; Kane et al., 2017; Killingsworth & Gilbert, 2010; Seli et al., 2018). Accordingly, scientific interest into what is generally referred to as “mind-wandering” remains high (Smallwood & Schooler, 2015). Current research has called for a more nuanced understanding of this construct and has highlighted the heterogeneity of related experiences (e.g., Seli et al., 2018; Wang et al., 2017). In particular, the differentiation of mind-wandering qualities such as content and form (Smallwood et al., 2016), and the exploration of specific contexts in which self-generated, task-unrelated thoughts occur, are argued to be important avenues to advance our understanding of the costs and benefits of different aspects of experience (Smallwood & Andrews-Hanna, 2013; Wang et al., 2017).

Studies routinely capture individuals’ internal experiences using experience sampling techniques (Csikszentmihalyi & Larson, 1987) both in the laboratory and in daily life. In the lab, researchers often exploit the ability to constrain task context to either induce mind-wandering (typically by keeping cognitive demands low; Smallwood, Nind, & O’Connor, 2009) or to detect the consequences of off-task thought using tasks which demand continuous external attention (e.g., McVay & Kane, 2009), or both (Turnbull et al., 2019). In contrast, daily life situations present us with more complex ecological contexts which may be less readily comparable to the lab situation. In contexts requiring high cognitive (e.g., attentional) capacities, mind-wandering has been related to disruptions of performance in complex tasks such as measures of intelligence (Mrazek et al., 2012) or reading (Schooler, 2004). However, studies show that ongoing experience is generally adjusted to current demands (Kane et al., 2007; Rummel & Boywitt, 2014). When demands are low, mind-wandering can be associated with beneficial outcomes such as facilitated prospection (Baumeister & Masicampo, 2010) or attenuated low mood through ‘mental breaks’ from monotonous occupation (Ruby, Smallwood, Engen, & Singer, 2013a). Mind-wandering has further been linked to creative thinking (see Fox & Beaty, 2019 for a recent review) and may aide creative problem solving (Baird et al., 2012), although conflicting results have been found (Smeekens & Kane, 2016). The importance of specifying the task context in understanding the links between internal experience and aspects of psychological functioning is known as the context regulation hypothesis (Smallwood & Andrews-Hanna, 2013).

Studies on the content of mind-wandering indicate a prospective bias (i.e., a tendency to engage in more future-directed thoughts; Smallwood & Schooler, 2015). This prospective bias is primarily regarded as reflecting the utility of future planning (Baird, Smallwood, & Schooler, 2011). It is thought to depend on autobiographical memories (Smallwood et al., 2011) and may afford the refinement of personal goals (Medea et al., 2018). Past-oriented thoughts, on the other hand, have been shown to follow unhappy moods (Smallwood & O’Connor, 2011). While prior accounts have suggested a general link of mind-wandering to unhappiness (Killingsworth & Gilbert, 2010), later findings taking the specific thought content into account have produced a more complex picture. Thus, the affective consequences of ongoing thought depend on its specific socio-temporal content both in the laboratory (Ruby et al., 2013a) and in daily life (Welz, Reinhard, Alpers, & Kuehner, 2018), and on the level of interest in the respective mind-wandering episodes (Franklin et al., 2013). Also, individual differences in thought content differentially relate to measures of emotional well-being (Andrews-Hanna et al., 2013). Taken together, the content regulation hypothesis formalises these differential accounts and stresses that the relation of psychological well-being and self-generated thought is dependent on an individual’s capacity to regulate their thoughts’ content (Smallwood & Andrews-Hanna, 2013).

Mind-wandering is an umbrella term for multiple conceptualizations of cognitive content (Seli et al., 2018). It shares overlap with perseverative cognition (Ottaviani, Shapiro, & Couyoumdjian, 2013), which refers to two particularly prominent types of thought patterns characterized by repetitive thinking with a negative focus on events in the past or future (rumination or worry; Brosschot, Gerin, & Thayer, 2006). Perseverative cognition has been associated with psychopathology (McLaughlin & Nolen-Hoeksema, 2011) and conceptually ties stress and its physiological correlates to somatic health risks (Ottaviani et al., 2016). Recent studies into mind–body interactions within the framework of stress have identified associations of thought content and stress-induced cortisol output both in the laboratory setting (Engert, Smallwood, & Singer, 2014) and daily life (Linz, Singer, & Engert, 2018). Specifically, more negative emotional and a pattern of less future- and self-focused thoughts were linked to increased cortisol levels both at rest and after an acute laboratory stress paradigm (Engert et al., 2014). In daily life, more past-focused thoughts were associated with increased cortisol in the absence of stress. When experiencing stress, however, more negative and more future-directed thoughts predicted increased cortisol release (Linz et al., 2018). In contrast to the lab study (Engert et al., 2014), and highlighting the context specificity of where thoughts are assessed, the social thought dimension was unrelated to subjective stress or cortisol in daily life (Linz et al., 2018).

Given the ubiquity of internal experience such as mind-wandering, studies have begun to explore its relationship in different contexts. The first study to investigate mind-wandering in both laboratory and daily life within the same population found the amount of reported mind-wandering to relate between contexts, concluding mind-wandering to be a stable cognitive characteristic (McVay, Kane, & Kwapil, 2009). Moreover, more off-task thoughts in the lab predicted more worrying in daily life (McVay et al., 2009). A longitudinal study by Ottaviani and Couyoumdjian (2013) found that the frequency of laboratory mind-wandering episodes correlated with those in daily life after more than year, suggesting a stable individual disposition. A more recent study questioned these results by showing that the mind-wandering rate in the laboratory only had a marginal relationship to the same measure in daily life, and this relationship was less robust than links with contextual predictors such as the current activity or affective state (Kane et al., 2017). Regarding the content of thoughts, little is known about how well laboratory findings generalise to daily life.

### Current study: aims and hypotheses

There were two aims to the current study. First, we analysed how both the content and focus of experience, i.e., specific content dimensions and the extent of off-task thinking, correlated within individuals from a controlled laboratory setting to daily life. Second, we explored moment-to-moment associations of subjective experience with both subjective and physiological stress markers, captured over 2 days of participants’ daily lives.

We aimed to compare the amount of off-task thinking in the laboratory and daily life based on the notion that the extent of mind-wandering is dependent on the demands of a given context (Kane et al., 2007), and recent evidence challenging the assumption of a consistent association between contexts (Kane et al., 2017). Emerging evidence suggests that a prospective bias is present in both the lab and daily life (e.g., Smallwood et al., 2009; Linz et al., 2018), and that it is most pronounced in situations with the lowest tasks demands. Future-directed thoughts have been shown to covary with self-focused thoughts (Ruby et al., 2013a), perhaps because of a reliance on underlying autobiographical, self-referential processes (Baird et al., 2011; Smallwood et al., 2011) which are particularly relevant to the individual (Stawarczyk, Cassol, & D’Argembeau, 2013). Accordingly, we aimed to identify whether the degree of future-directed and self-focused thought is particularly stable between contexts. Furthermore, as negative thought patterns such as rumination or worry are not only repetitive but also tend to occur habitually (Watkins, 2008), the emotional valence of thoughts may generalize well from one context to the other, particularly so when negative.

We expected subjective stress to be associated with momentary demands and thus to inversely relate to off-task thinking. Regarding thought content, we expected stress to be primarily related to negative (and inversely to positive) thoughts, as seen in Linz et al. (2018). Perseverative cognition implies a self-referential component of thought irrespective of the temporal focus (Brosschot, 2010), which may thus be associated with higher levels of subjective stress. Finally, based on our previous findings, we hypothesized a link between negative thought content and cortisol (Engert et al., 2014; Linz et al., 2018).

## Methods

### Participants

Ninety-three participants (47 women, age mean ± SD = 25.4 ± 3.9 years, age range 18–35 years) provided daily life experience sampling data for the present study. Repeated unavailability or insufficient compliance with the study protocol lead to the exclusion of six initially eligible participants, who had previously taken part in the laboratory testing session (reported in Engert et al., 2014). The majority of participants were students (77%), 14 (15%) were employed, eight (9%) held no job. A higher education degree was held by 27% of participants, 69% held a higher education entrance qualification (e.g., high-school diploma). Compliance with eligibility criteria was ascertained in a structured telephone interview targeting current and recent history of psychological and physiological disorders as well as medication and drug consumption. To avoid confounding effects on both subjective measures and cortisol levels, exclusion criteria were regular smoking or recreational drug use, chronic illness, current psychological disorder and medication intake affecting the HPA axis. To limit effects of sex hormones on cortisol levels (Kajantie & Phillips, 2006), female participants did not use hormonal contraceptives and were tested in the luteal phase of their cycle. The study was approved by the Research Ethics Board of Leipzig University (ethics number: 360-10-13122010). Participants gave written informed consent, could withdraw from the study at any time and received financial compensation.

### Procedure

Participants completed 2 days of experience sampling in daily life. In addition, the majority of participants (*N* = 88) had previously taken part in a laboratory study assessing mind-wandering in relation to a psychosocial stress paradigm (reported in Engert et al., 2014), from which the baseline mind-wandering measurement was obtained for comparison with current daily life mind-wandering parameters. Daily life sampling was completed within the week following the lab testing session. Participants were advised to choose 2 regular consecutive weekdays representative of their daily life routines (Monday/Tuesday, Wednesday/Thursday or Thursday/Friday, depending on participant availability). All experience sampling data were gathered using mobile devices, which were equipped with a custom inhouse software app. At each designated sampling time point, the app prompted the respective subjective experience questionnaires and reminded participants to take a saliva sample. To ensure proficiency in handling the mobile device and self-administering saliva samples, participants received an introductory training before data collection. Saliva sampling started immediately upon free awakening (while still in bed). Further sampling times were 30 and 60 min later, as well as at 12:00, 3:00, 6:00 and 9:00 pm. Experience sampling questionnaires overlapped with the cortisol sampling schedule, except for the wakeup sample for which no experience sampling was recorded. Sampling times during the day had a margin of fluctuation (± 15 min of the fixed interval) to avoid complete predictability. The thought sampling procedure of the laboratory testing is described in detail in Engert et al. (2014). In short, participants followed a routine paradigm for sampling self-generated thoughts (e.g., Smallwood et al., 2011), which consisted of repeated thought content probes intermittent of two cognitive tasks repeatedly employed in studying mind-wandering (see Engert et al., 2014 and below for details).

### Measures

#### Daily life subjective experience

Throughout the day, participants provided information on their momentary subjective experience including their content of thought and the extent of off-task thinking, affect and arousal as well as the occurrence of subjective stress.

*Thought content and extent of mind*-*wandering* In analogy to the laboratory testing (Engert et al., 2014), participants indicated at each sampling time point their current thought content in six separate content items (on a scale of 1–10): positive and negative (valence), future-directed and past-directed (temporal), and self-focused and other-oriented (social). In addition, they rated the extent to which their current thoughts were unrelated to their current task (off-task, on a scale from 1 to 10) as a proxy for the mind-wandering extent. Laboratory mind-wandering was sampled during two counterbalanced sessions of both the choice reaction time task (CRT) and the working memory task (WM), two tasks frequently used in previous laboratory mind-wandering studies (Engert et al., 2014; Smallwood, Ruby, & Singer, 2013; Smallwood et al., 2011).

*Affect and arousal* Concurrent affect and arousal were obtained using the Affect Grid (Russel, Weiss, & Mendelsohn, 1989), a single item scale assessing the dimensions pleasure-displeasure and arousal-sleepiness (both on a scale from 1 to 9) with adequate reliability, convergent and discriminant validity.

*Subjective stress* At each sample, participants further indicated how stressed they felt (on a visual analogue scale ranging from 1 to 10).

#### Salivary cortisol

Saliva was sampled into Salivette collection devices (Sarstedt, Nümbrecht, Germany). Participants were instructed to place the collection swabs in their mouths and to refrain from chewing for 2 min. They were asked to refrain from any oral intake during the 10 min before sampling, and otherwise followed their normal daily routine. Participants were instructed to store the salivettes in the freezer as soon as their daily routines permitted. Upon return to the laboratory, salivettes were stored at − 30 °C until assay (at the Department of Biological and Clinical Psychology, University of Trier, Germany). Cortisol levels (expressed in nmol/l) were determined using a time-resolved fluorescence immunoassay with intra-/inter-assay variabilities of < 10%/12%.

### Statistical analysis

We applied principal component analysis with varimax rotation to decompose all daily life thought samples across participants into components reflecting patterns of covarying thought content dimensions (see Engert et al., 2014 for analogous decomposition of laboratory samples). To analyse associations of mind-wandering content between the two contexts, we used multiple correlations to relate averages of each content between daily life and laboratory. Statistical significance in this analysis was controlled for seven comparisons via Bonferroni correction and alpha threshold lowered to (0.05/7) = 0.007.

To analyse moment-to-moment associations of both subjective- and physiological measures of stress with subjective experience including thought content, we built two mixed-effects models with (1) subjective stress and (2) salivary cortisol as dependent variables of all available samples across days and subjects. Both models included a random intercept for subject. Since cortisol secretion follows a diurnal rhythm and is influenced by individual characteristics (Kudielka, Gierens, Hellhammer, Wust, & Schlotz, 2012), we included age, sex, time of sample as well as wakeup time as covariates in Model 2. In this model, we excluded all samples associated with the cortisol awakening response (wakeup, 30 min and 60 min samples) from analysis, since it has been shown to represent a distinct feature of the cortisol circadian rhythm (Wilhelm, Born, Kudielka, Schlotz, & Wüst, 2007) not readily comparable to samples later during the day (Stadler et al., 2015). Both models are specified below in Raudenbush and Bryk (2002) notation:

Model 1:

Level 1: *Y*_**s**di_ = *π*_0di_ + *π*_1di_ (off-task) + *π*_2di_ (negative) + *π*_3di_ (positive) + *π*_4di_ (self) + *π*_5di_ (other) + *π*_6di_ (future) + *π*_7di_ (past) + *π*_8di_ (affect) + *π*_9di_ (arousal) + e_tdi_
Level 2: *π*_0di_ = *β*_00i_ + *u*_0di_
Level 3: *β*_00i_ = *γ*_000_ + *r*_00i_

Model 2:

Level 1: *Y*_**s**di_ = *π*_0di_ + *π*_1di_ (time) + *π*_2di_ (stress) + *π*_3di_ (off-task) + *π*_4di_ (negative) + *π*_5di_ (positive) + *π*_6di_ (self) + *π*_7di_ (other) + *π*_8di_ (future) + *π*_9di_ (past) + *π*_10di_ (affect) + *π*_11di_ (arousal) + *e*_tdi_
Level 2: *π*_0di_ = *β*_00i_ + *β*_01i_ (awakening time) + *u*_0di_
Level 3: *β*_00i_ = *γ*_000_ + *γ*_001_ (sex) + *γ*_002_ (age) + *r*_00i_

Taking into account the hierarchical structure of the data with multiple samples (Level 1) nested into days (Level 2) and subjects (Level 3), the respective dependent variable (subjective stress or cortisol level) was predicted by the intercept (*π*_0di_), all measures of momentary subjective experience on level 1, day-specific predictors (e.g., awakening time in model 2) on level 2 and individual predictors (e.g., sex in model 2) on level 3. Both models’ residuals displayed only very negligible deviations from normal distribution. Further checks on potential multicollinearity of the models’ predictors were negative: all variance inflation factors were < 0.15, indicating the models’ efficacy in assessing the unique influence of all predictors on the respective dependent variable. Estimates reported are restricted maximum likelihood marginal estimates using an unstructured covariance structure. Raw cortisol values were ln-transformed before analysis. All analyses were performed using *R* version 3.5.1 (R Core Team, 2018) and the packages *lme4*, *car and psych*. Unless stated otherwise, significance was set to a level of *p* (alpha) < 0.05.

## Results

### Descriptive statistics

Compliance with the study protocol was overall satisfactory: Regarding electronic probes of daily life thoughts, 1043 (93.4% of 1116 total) probes were completed, while 1219 (93.6% of 1302 total) cortisol samples entered analysis. Table 1 presents descriptions of subjective experience samples in daily life. We found that thoughts were more future- than past-directed [*t*(1042) = 21.847, *p* < 0.001] and more positive than negative in valence [*t*(1042) = 23.515, *p* < 0.001]. The social dimension of thought (self vs. other) was balanced [*t*(1042) = 0.81613, *p* > 0.4]. Ratings of the extent to which thoughts were off vs. on current task indicated that participants were more frequently off-task than on-task (147 out of 1043 probes [14%] were completely off-task; 104 out of 1043 probes [10%] completely on-task). Reported levels of subjective stress were on average low; specifically, in 455 out of 1043 probes (43%) participants reported the lowest possible stress rating (on a continuous scale from 1 to 10).

**Table 1:** Descriptive statistics of daily experience samples

	Mean (range)	SD	*n*	*t*, *p*(*t*)
Off-task	5.47 (1–10)	2.99	1043	
Negative	2.5 (1–10)	2.17	1043	23.515, *p* < 0.001
Positive	5.29 (1–10)	2.53	1043	
Self	4.54 (1–10)	2.58	1043	0.81613, *p* = 0.414
Other	4.45 (1–10)	2.87	1043	
Future	5.16 (1–10)	2.81	1043	21.847, *p* < 0.001
Past	2.72 (1–10)	2.48	1043	
Valence (affect grid)	6.32 (1–9)	1.74	1148	
Arousal (affect grid)	4.78 (1–9)	2.14	1148	
Subjective stress	2.65 (1–10)	2.03	1043	

### Relation of laboratory and daily life mind-wandering: extent and content

**Fig. 1:**
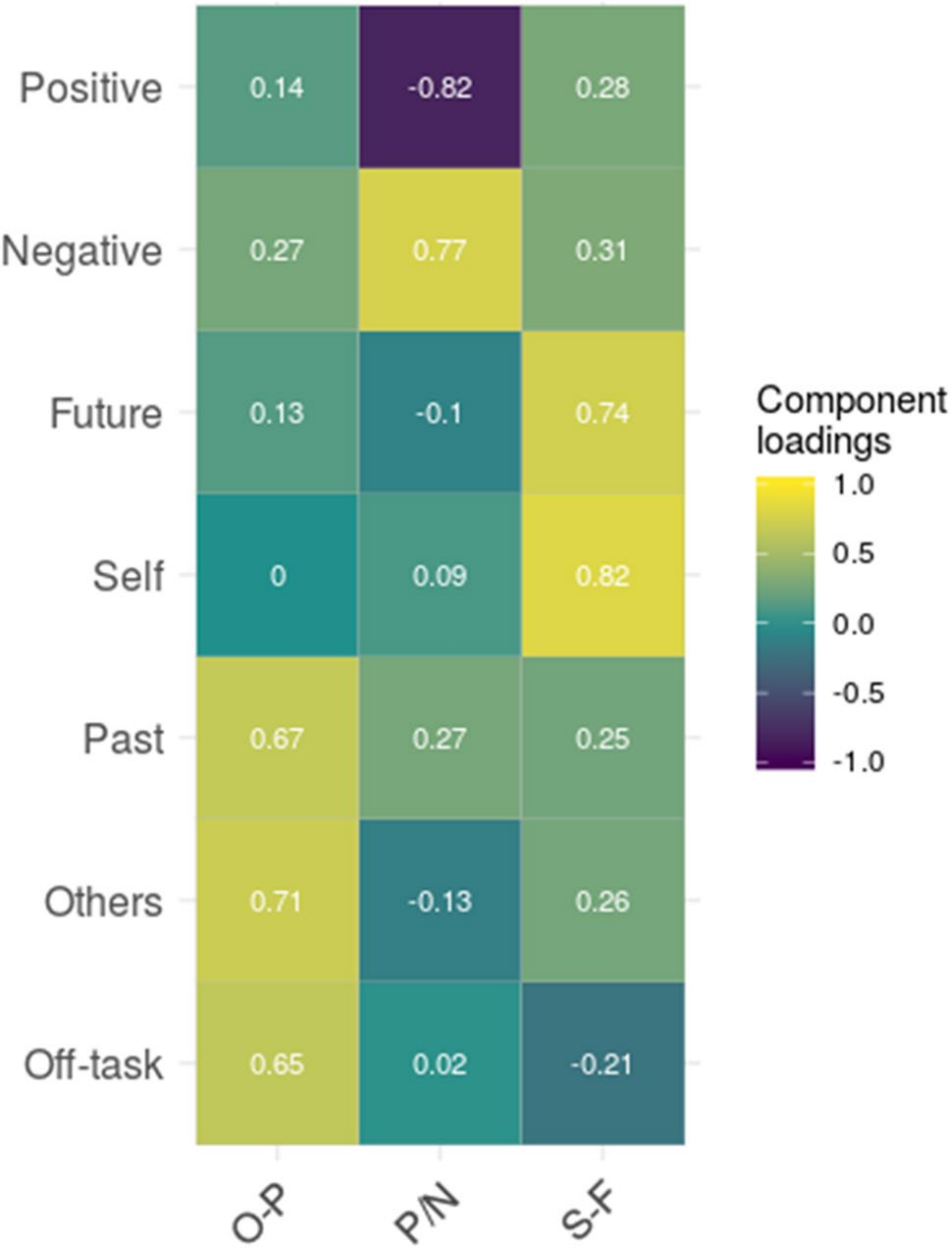
Principal components and respective rotated component loadings of daily life thought probes

In a first step, we applied principal component analysis (PCA) with varimax rotation to all daily life thought samples across all participants. Figure 1 shows loadings of the three components derived from the thought content samples indicating similar results as have been found in previous studies (Ruby et al., 2013a; Ruby, Smallwood, Sackur, & Singer, 2013b) and a largely overlapping set of participants as tested in the laboratory setting (Engert et al., 2014). The three components accounted for a cumulative 63% of variance and reflected (a) social–temporal thoughts directed at oneself- and the future (S–F), (b) off-task and social-temporal thoughts directed towards others and the past (O–P), and (c) thoughts both positive and negative in valence (P/N).

**Fig. 2:**
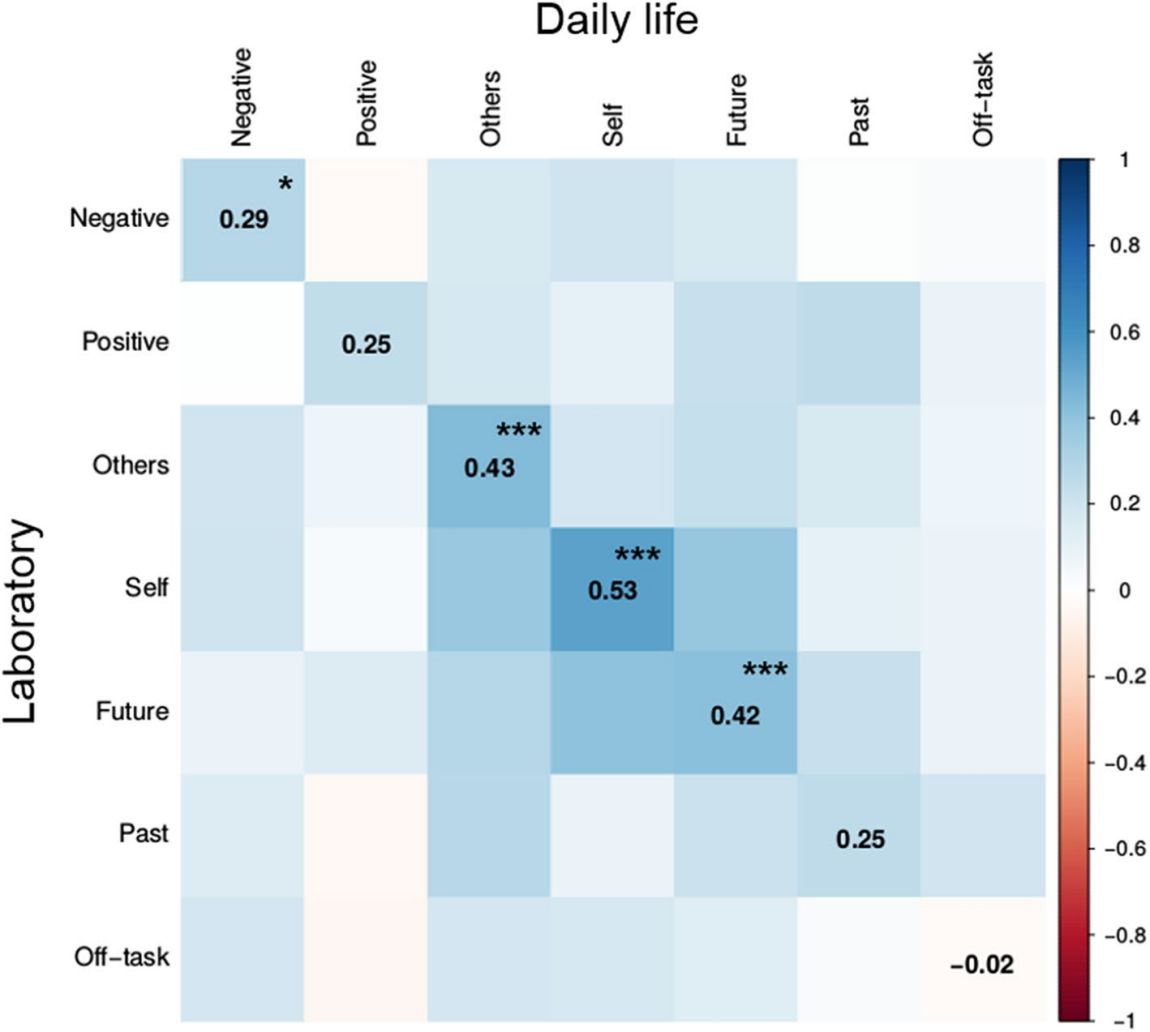
Correlations of mind-wandering content between daily life and laboratory context

To investigate how the laboratory thought content translates to daily life, we calculated multiple within-subject correlations between the averages of specific thought content dimensions of the two contexts. Table 2 and Fig. 2 show the respective correlations. The social dimension (both self- and other-directed thoughts), future-directed thoughts, and negative thoughts showed highly significant correlations between contexts. Correlations of positive thoughts or past-directed thoughts between laboratory and daily life were not significant after Bonferroni correction for multiple comparisons. The extent to which participants’ thoughts were off-task in the laboratory session and in daily life were also not associated.

**Table 2:** Correlations of respective thought content between laboratory and daily life context

	*r*	*t*	*df*	*p*
Off-task	− 0.023	− 0.214	86	0.830
Negative	0.289	2.808	86	0.006*
Positive	0.248	2.381	86	0.019
Self	0.530	5.809	86	<0.001***
Other	0.430	4.422	86	<0.001***
Future	0.419	4.285	86	<0.001***
Past	0.252	2.420	86	0.018

*p < 0.007; ***p < 0.00014

### Associations of daily life subjective experience with markers of stress

To investigate the relation of measures of subjective experience (thought content, extent of mind-wandering, affect, and arousal) with measures of stress, we ran two separate models assessing moment-to-moment associations of subjective experience with (1) subjective stress and (2) cortisol as dependent variables.

Model 1 examined the association of subjective experience with subjective stress. Estimates of Model 1 parameters are displayed in Table 3. More subjective stress was associated with more negative and less positive thoughts, more future- and less past-directed thoughts as well as more task focus (i.e., less mind-wandering) overall. In addition, subjective stress was linked to higher levels of arousal.

**Table 3:** Estimates for Model 1 (predictors of subjective stress)

Fixed effects	*B* (SE)	CI	*p*
(Intercept)	2.65 (0.103)	2.45 to 2.85	< 0.001
Off-task	− 0.05 (0.175)	− 0.1 to − 0.01	0.019
Negative	0.32 (0.035)	0.25 to 0.39	< 0.001
Positive	− 0.12 (0.029)	− 0.18 to − 0.07	< 0.001
Self	− 0.01 (0.029)	− 0.07 to 0.05	0.71
Other	− 0.02 (0.026)	− 0.07 to 0.03	0.45
Past	− 0.09 (0.031)	− 0.15 to − 0.02	0.006
Future	0.06 (0.026)	0.01 to 0.11	0.018
Affect	− 0.07 (0.040)	− 0.15 to 0.01	0.084
Arousal	0.09 (0.034)	0.03 to 0.16	0.007

Random effects	Variance	*SD*
Individual (intercept)	0.495	0.70
Day (intercept)	0.154	0.39
Residual	2.749	1.66

Model 2 examined the association of salivary cortisol levels with all subjective experience measures. Table 4 displays the estimates of Model 2 parameters including covariates sampling time, time of wakeup and sex. Sampling time was the strongest predictor of cortisol levels (*β*_30_ = 0.001, *t* = 3.05, *p* = 0.003) such that lower cortisol levels were associated with longer time between wakeup and a respective sample. Furthermore, subjective stress and earlier time of awakening were linked to higher cortisol levels. All other measures of subjective experience and sex were unassociated with concurrent cortisol levels.

**Table 4:** Estimates for Model 2 (predictors of cortisol)

Fixed effects	*B* (SE)	CI	*p*
(Intercept)	1.73 (0.237)	1.28 to 2.19	< 0.001
Off-task	− 7.37 (0.010)	− 0.01 to 0.03	0.47
Negative	6.83 (0.035)	− 0.02 to 0.04	0.68
Positive	− 3.68 (0.013)	− 0.03 to 0.03	0.78
Self	9.72 (0.013)	− 0.01 to 0.03	0.46
Other	− 1.04 (0.012)	− 0.03 to 0.01	0.37
Past	1.98 (0.014)	− 0.00 to 0.05	0.16
Future	1.11 (0.012)	− 0.01 to 0.03	0.36
Affect	1.69 (0.018)	− 0.01 to 0.05	0.34
Arousal	2.07 (0.015)	− 0.01 to 0.05	0.18
Subjective stress	3.37 (0.016)	− 0.01 to 0.06	0.03
Sex	1.07 (0.092)	− 0.07 to 0.28	0.25
Age	− 4.57 (0.012)	− 0.02 to 0.02	0.99
Time	− 1.24 (0.008)	− 0.14 to − 0.11	< 0.001
Awakening time	− 1.24 (0.000)	− 0.00 to − 0.00	0.005

Random effects	Variance	*SD*
Individual (intercept)	0.096	0.31
Day (intercept)	0.008	0.09
Residual	0.330	0.57

## Discussion

Research in the mind-wandering domain has highlighted the importance of both context and content when assessing the characteristic features of ongoing thought (Smallwood & Andrews-Hanna, 2013). In particular, emerging evidence has highlighted the need to understand the extent to which laboratory findings generalize to more complex, ecologically valid contexts in daily life (Kane et al., 2017). Our study provides evidence on (1) how different aspects of ongoing thought differentially translate from the laboratory to daily life and (2) how both subjective stress as well as associated levels of the HPA axis end hormone cortisol relate to thought content and other subjective experiences such as affect and arousal in everyday life. We found that only certain aspects of ongoing thought were correlated across situations. Individual variation in social thoughts (about oneself and others), future-directed and negative thoughts displayed high stability across the lab and daily life, while past-directed and positive thoughts were less stable. Importantly, the degree of off-task thinking did not transfer from lab to daily life. Together, our findings highlight that the transferability of experiential reports from the lab to daily life is content-specific. They suggest that not only for *whom* and *when the mind wanders* (Kane et al., 2017), but also *where* it wanders may vary between laboratory and daily life.

We found no correlation in reports of off-task thinking between different contexts, and so our results diverge from those by McVay et al. (2009) and Ottaviani and Couyoumdjian (2013), and suggest an even less robust association than the well-powered study by Kane et al. (2017). A simple explanation for this divergence could be the way internal experience was measured. In our study, we separated multiple features of ongoing thought (task focus, temporal and social content, and affective qualities), while others have used a method in which these features were combined (i.e., concepts like perseverative cognition or everyday worries entail combinations of features of experience such as self-focus and affective content, that our study measured individually). On considering these issues, our methods of conceptualizing experience along separate dimension is possibly more conservative, because it allows us to distinguish aspects of experience that do generalize (i.e., self, future and social foci) from those that do not (being off-task). We also measured off-task thinking in a continuous manner, while others have operationalized mind-wandering dichotomously (Kane et al., 2017; McVay et al., 2009), and recent evidence suggests substantial differences in daily life off-task estimates depending on whether dichotomous or continuous rating options are employed (Seli et al., 2018).

In addition, the level of similarity between laboratory and daily life contexts likely contributes to diverging results. Ottaviani and Couyoumdjian (2013) differentiated mind-wandering from perseverative cognition and distraction, and found that the frequency of mind-wandering episodes in the laboratory predicted daily life mind-wandering a year later. Importantly, their laboratory session featured an ecological stress induction, and arguably, laboratory conditions more closely corresponding to daily life situations (where naturally occurring stressors are common) should increase correlations of mind-wandering rates.

Our study adds to an emerging picture regarding the complex links between stress and ongoing experience in daily life. We found higher subjective stress was linked to fewer off-task thoughts, a greater future focus and more negative cognition, a pattern suggesting that, in daily life, stress is often associated with the need to act. Our findings are different from those of McVay et al. (2009), who found an increased occurrence of task-unrelated thought in stressful situations and from those of Croswell and colleagues, who found unpleasant and neutral mind-wandering was associated with higher chronic stress as well as retrospective reports of daily stress (Crosswell, Coccia, & Epel, 2019). However, Kane et al. (2017) found that the mind-wandering probability was not significantly predicted by how stressful a current situation was experienced. We hypothesize that in the current study, moment-to-moment associations of stress with future-directed thoughts may reflect goal-directed planning, and potentially involve proactive efforts to alleviate ongoing or anticipated task strain. Past-directed thoughts, on the other hand, may have emerged from the ‘luxury’ of not having immediate tasks at hand (or not anticipating those). This rationale is supported by the pattern of our PCA analysis, which revealed covariation of past-focused and off-task thoughts. Negative (and, inversely, positive) thought content was the strongest predictor of subjective stress and remarkably, negativity/positivity of thought was a much stronger predictor of stress than (negative/positive) affect, which mirrors our recent daily life findings of an association of negative thoughts and cortisol levels (Linz et al., 2018). We did not find self- or other-focused thoughts to be linked to subjective stress. While a (negative) self-focus is an essential characteristic of perseverative cognition (Brosschot et al., 2006), our recent findings suggested a rather low prevalence of perseverative cognition in the daily life experience of healthy subjects (Linz et al., 2018). Conceivably, in healthy individuals, most stressful situations in daily life do not take place in isolation, but rather arise from social situations including both ourselves and others. Overall, emerging evidence illustrates links between stress and ongoing thought, and our data reinforce the need to recognize the complexity of this relationship. Future studies should profit from measuring multiple features of thought content when investigating the role of stress in daily life subjective experience.

The biomarker cortisol was not associated with any measure of subjective experience. Several reasons may explain why a link of cortisol and thought content may be less easily detectable in daily life than during a stress paradigm in the laboratory (Engert et al., 2014). Daily life stressors (and accompanying fluctuations in cortisol) are likely less pronounced than a full-blown laboratory stressor. Furthermore, while cortisol sampling in the laboratory is well controlled, samples in daily life are self-administered, and likely less reliable. Moreover, studies in ecologically valid environments are inherently noisier than controlled laboratory settings: a large proportion of variance in diurnal cortisol levels, for example, is explained by contextual factors (Kudielka et al., 2012). While we controlled for the biggest source of variance in diurnal cortisol levels, the respective time of each sample (Kudielka et al., 2012), other potential influences arising from the varying circumstances of everyday life (e.g., food or caffeine intake, physical activity, social interactions) may have obscured potential relations. In comparison to our recent finding of an interaction of stress and thought content in predicting cortisol in daily life (Linz et al., 2018), factors such as a different modelling approach, a smaller sample size and a limited age range of the investigated sample may have hindered corroborating evidence in the current study.

Several limitations of the present study need to be taken into account. First, it would have been advantageous to assess additional mind-wandering characteristics such as the form of thoughts (Smallwood et al., 2016) especially given recent evidence on the role of the default mode network in this feature of experience (Sormaz et al., 2018). On the same note, research has argued that intentional and unintentional mind-wandering are dissociable cognitive experiences (Seli, Risko, Smilek, & Schacter, 2016), which have been shown to differentially relate to the content and potential consequences of mind-wandering (Seli, Beaty, Marty-Dugas, & Smilek, 2019; Seli, Ralph, Konishi, Smilek, & Schacter, 2017). Likewise, we did not assess intrusiveness, repetitiveness, ruminative or worrysome qualities of thoughts, which would have enabled us to operationalize perseverative cognition in a more direct manner. Measuring personality traits such as trait anxiety or levels of depression may have shed light on interindividual differences mediating the relationship between thought content and stress. Because daily life episodes can be ambiguous regarding a current task or even be task-free (see Murray, Krasich, Schooler, & Seli, 2019 for an in-depth discussion), relying on a task-related operationalization of mind-wandering imposes general methodological challenges in daily life studies. Finally, cortisol measurement in ambulatory settings should be treated with some caution, unless using ways to objectively verify participants’ adherence to the sampling protocol (Stadler et al., 2015).

Before concluding, it is worth considering how our results can aid our understanding of how to link experience from the laboratory to daily life. Together, our data suggest patterns of off-task thinking are uncorrelated across situations, and in daily life are associated with lower levels of stress. Based on these data, a potential reason for why measures of on-task experience do not always generalize across contexts may be that in daily life, individuals are more likely to choose the actions they perform, and arguably are also more motivated to perform these tasks. In particular, highly constrained tasks are likely less common in daily life, but conceivably allow greater freedom to consider other topics and may have greater alignment to an individual’s goals (Murray et al., 2019). More generally, studies suggests that individuals’ off-task thoughts often have social features, and while laboratory task contexts often do not entail these social stimuli, they are frequently present in the real world. Self-generated thoughts are often assumed to reflect an individual’s current concerns (e.g., Klinger & Cox, 1987), a perspective that is supported by recent evidence implicating the dorsolateral pre-frontal cortex in the prioritisation of off-task thoughts in situations of low task demands (Turnbull, Wang, Murphy, et al., 2019). This prioritisation view of ongoing thought gains support from studies showing that the frequency of mind-wandering in the laboratory is closely linked to participants’ motivation (Seli, Cheyne, Xu, Purdon, & Smilek, 2015; Seli, Wammes, Risko, & Smilek, 2016), and that individuals can flexibly modulate their mind-wandering rates depending on upcoming demands (Seli et al., 2018; Turnbull, Wang, Schooler, et al., 2019; Turnbull, Wang, Murphy, et al., 2019). Together, these lines of evidence suggest that while thoughts with personally relevant content may be distractions in the lab (Stawarczyk, Majerus, Maj, Van der Linden, & D’Argembeau, 2011; Unsworth, McMillan, Brewer, & Spillers, 2012), they may be more closely aligned to opportunities to act in everyday life. Accordingly we suggest that if researchers want to approximate patterns of experience that correspond to those occuring in the real world, they should either assess experience outside of the lab, or measure patterns of experience in laboratory contexts with greater ecological validity and/or personal significance. We note that our data do not invalidate the exploration of patterns of ongoing experience in laboratory conditions. Instead, our study highlights the need to explicitly consider the boundary conditions of this approach when attempting to generalize from the lab to daily life.

In conclusion, we find that measures of ongoing thought differentially translate from lab to daily life. Our findings suggest that patterns of off-task thinking may not be reliably inferred from laboratory data, while specific content of thoughts, such as its social or episodic features, does transfer. Furthermore, we show a link between subjective stress and distinct thought content in daily life, with greater subjective stress linked to more on-task thoughts with a negative future focus. Taken together, our results add to a growing body of research emphasizing the heterogeneous nature of the wandering mind. We propose that findings in the mind-wandering domain should be carefully interpreted regarding their applicability to life outside the laboratory.

## Data Availability

The datasets analysed during the current study are not publicly available due lacking participant consent for data-sharing with third parties (according to our current General Data Protection Regulation, GDPR), but are available from the corresponding author on reasonable request.
